# Risk factors of early death in pediatric hemophagocytic lymphohistocytosis: Retrospective cohort study

**DOI:** 10.3389/fped.2022.1031432

**Published:** 2022-10-21

**Authors:** Lijun Zhang, Lei Dai, Deyuan Li

**Affiliations:** ^1^ Department of Pediatrics, West China Second University Hospital, Sichuan University, Chengdu, China; ^2^ Key Laboratory of Birth Defects and Related Disease of Women and Children (Sichuan University), Ministry of Education, Chengdu, China; ^3^ State Key Laboratory of Biotherapy and Cancer Center, West China Hospital, Sichuan University and Collaborative Innovation Center for Biotherapy, Chengdu, China

**Keywords:** hemophagocytic lymphohistiocytosis, children, early death, risk factor, heart failure

## Abstract

**Background:**

Hemophagocytic lymphocytosis (HLH) is a rare life-threatening hyperinflammatory syndrome in which early mortality remains high in patients with HLH.

**Methods:**

We retrospectively collected the medical records of all pediatric patients diagnosed with HLH at the West China Second Hospital of Sichuan University between January 2014 and December 2020. Collect demographic, laboratory, clinical, genetic profiles, treatment information and perform statistical analysis from records. Risk factors for death 30 days after admission were evaluated using a multivariable logistic regression model.

**Results:**

A total of 110 pediatric HLH patients were enrolled. The median age of patients was 44 months (IQR 23-100.5) and 62 (56.4%) in males. The 30-day mortality rate for admission to this cohort was 34 (30.9%). Multivariate logistic regression analysis showed that heart failure (OR = 13.389, 95% CI, 1.671–107.256, *p *= 0.015) and hypoproteinemia (OR = 4.841, 95% CI, 1.282–18.288, *p *= 0.020) were associated with increased early mortality in children with HLH.

**Conclusions:**

These identified risk factors may help clinicians stratify patients with HLH and develop targeted treatment strategies. More research is needed to explore the best treatment strategies for patients with HLH to reduce early mortality in patients with HLH.

## Introduction

Hemophagocytic lymphocytosis (HLH) is a rare hyper-inflammatory syndrome characterized by an overactive immune system leading to hypercytokinemia and multi-organ dysfunction. HLH is divided into primary HLH (pHLH), which is due to genetic defects and usually familial, and secondary HLH (sHLH), which is due to infection (e.g., Epstein-Barr virus, EBV), tumors and other underlying factors (1–4). Both can be detected at any age and can be life-threatening if left untreated and effective (3, 5).

Clinical features of HLH include persistent hyperthermia, splenomegaly, cytopenia, hypertriglyceridemia, hyperferritinemia, and elevated levels of soluble CD25 (sIL-2r) (6, 7). In addition, the central nervous system (CNS) is often affected, manifested by epilepsy and cognitive dysfunction (8, 9). Laboratory tests include specific bone marrow examination and nonspecific expression of high cytokines (e.g., interleukin-6 [IL-6], interleukin-12 [IL-12], and tumor necrosis factor-a [TNF-a]) (10). Diagnosis of HLH is not difficult, but needs to be differentiated from other highly inflammatory diseases. It is important to note that chemotherapy and/or immunotherapy are often required due to the difficulty of distinguishing between pHLH and sHLH at the beginning of the diagnosis (11). In our cohort, the majority of patients belong to sHLH (especially sHLH due to EBV infection).

About 80 years ago, Scott and Robb-Smith first described the disease (12). In the time that followed, more HLH was reported, however, HLH was notorious for its high mortality rate. For example, about 10 years ago, there were reports that the 180-day survival rate for sHLH was only 26% (13). Happily, with the continuous improvement of diagnosis and treatment options, its long-term survival rate has improved significantly. Recently, in a cohort study of HLH patients in Yoon et al., the 5-year survival rates for sHLH induced by different etiologies (e.g., EBV infection, autoimmune disease, other infections, or unexplained causes) were 25.1%, 82.4%, 78.7%, and 55.5%, respectively (14). Still, the early high mortality rate of HLH is significant, and it may be of great significance that research can predict risk factors for early death from HLH. That's why we conducted this study. By reviewing the medical records of a tertiary children's hospital in southwest China and following up HLH patients included in the study, we described the clinical parameters of these patients and analyzed the risk factors associated with 30-day mortality.

## Materials and methods

### Patients

This is a retrospective cohort study. Data collection and graphical analysis took place between March 2022 and July 2022. We reviewed all children with HLH in the Information Management System (HIS) of West China Second Hospital of Sichuan University between January 2014 and December 2020. The inclusion criteria were: (1) age less than 18 years, (2) hospitalized patients (ICD-10) code D76.1 (hemophagocytic lymphohistocytosis) or D76.2 (hemophagocytic syndrome, infection-related). Exclusion criteria were: (1) children who did not meet the HLH-2004 diagnostic criteria, (2) not a first admission, (3) were incomplete, (4) lost follow-up, and (5) children who had been hospitalized for less than 24 h.

### Definitions

HLH diagnostic criteria confirmed using HLH-2004 (6, 15). Briefly, molecular diagnostics that are HLH compliant (e.g., PRF1, UNC13D, STX11, STXBP2, Rab27α, SH2D1A, BIRC4) and/or meet 5 of the following first 7 diagnostic criteria (due to the availability of soluble CD25 in our hospital): (1) Fever (≥38.5°C), (2) splenomegaly, (3) Cytopenia (at least one cytopenia) (Hb < 90 g/L [neonatal <100 g/L], platelet <100  ×  109/L, absolute neutrophil count [ANC] < 1.0 × 109/L), (4) hypertriglyceridemia (fasting triglycerides ≥2.0 mmol/L) and/or hypofibrinemia (fibrinogen ≤1.5 g/L), (5) hemophagocytosis (bone marrow, spleen, lymph nodes, or liver), (6) ferritin ≥500 µg/L, (7) low/absent natural killer (NK) - cell activity (<10 solute units), (8) soluble CD25 (sIL-2r) (≥2,400 U/ml, or average >±2SD).

### Data collection

Data was collected from medical records by two trained investigators. We collected demographic characteristics, laboratory metrics at admission, genetic testing data from patients, and treatment strategies during hospitalization. Common complications including multiple organ dysfunction syndrome (MODS), respiratory failure, heart failure, acute liver failure, acute kidney injury (AKI), acute respiratory distress syndrome (ARDS), sepsis, coagulopathy, severe pneumonia, electrolyte abnormalities, hypoproteinemia, liver dysfunction, and hyperlactatemia were also collected. Among them, respiratory failure is defined as PaO_2_ < 60 mm Hg in ambient air. Heart failure is defined in line with guidelines published by the European Society of Cardiology in 2016 (16). AKI is defined in line with the clinical practice guidelines published by KDIGO 2012 (Kidney Disease: Improving Global Outcomes): serum creatinine levels increased by a factor of 1.5 from baseline (17). Hypoproteinemia is defined as: serum albumin <30 g/L. Other complications are diagnosed according to guidelines and/or consensus.

### Statistical analysis

For continuous variables, we expressed them with median and interquartile range (IQR) and used the Mann–Whitney *U* test for between-group comparisons. For categorical variables, absolute values and percentages were expressed, and Fisher's exact test was used for between-group comparisons. The investigation of 30-day mortality prediction in patients with HLH was initially conducted by univariate analysis between the two groups to identify all potential associated factors. After binary analysis, the independent variables with *p*-value less than 0.05 were selected and entered into the multivariable Logistic regression analysis model. Survival curves were estimated by the Kaplan-Meier method. The survival time of the two groups was compared using the log-rank test. All statistical analyses were performed using SPSS (IBM SPSS Statistics for Windows, Version 26.0; IBM corp., Armonk, NY). Statistical significance for all tests was two-tailed *p* < 0.05. There were no trials of any specific treatment or preventive intervention. This study was approved by the Ethics Committee of the West China Second Hospital of Sichuan University (approval number: 2022-127).

## Results

**Figure 1:**
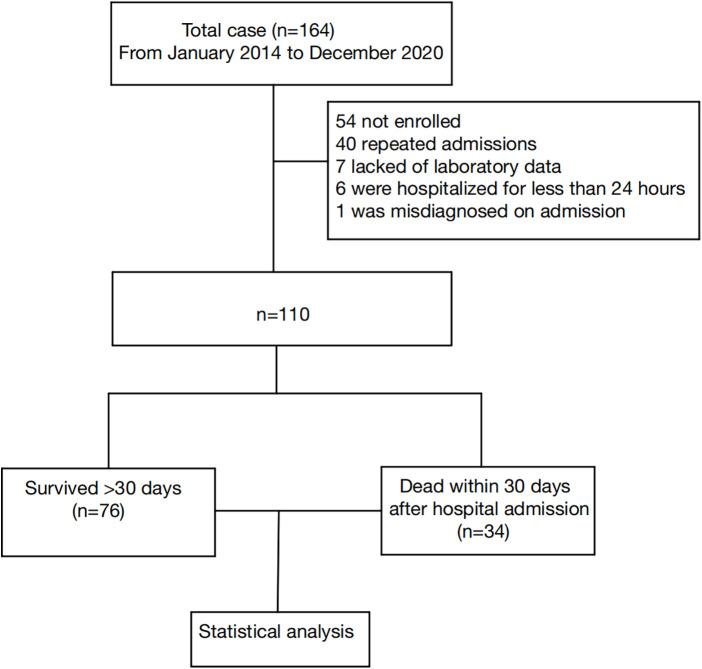
Study flow chart detailing the inclusion and exclusion of cases. Data collection flow chart for a total of 164 patients, with 110 final samples and 54 excluded cases.

A total of 164 patients passed the inclusion criteria. After an initial review, 54 patients were excluded, including 40 patients with repeat admissions, 7 patients with lack of primary data, 6 patients with a hospital stay of less than 24 h, and 1 patient not meeting the HLH-2004 diagnosis standard. The final analysis included 110 patients (Figure 1).

### Clinical data comparison between survival and death groups

As shown in Table 1, the median age of patients at diagnosis was 44 months (IQR 23.0–100.5). Of these, 62 patients (56.4%) were male. Our records show no significant differences between the two groups in terms of gender, age, weight. Of the 110 patients, 109 were admitted to the ICU, 34 (30.9%) died within 30 days of admission, and the 30-day OS was 69% (76/110). 109 (99.1%) patients developed fever and 90 (81.8%) developed splenomegaly. Strikingly, all patients developed hyperferritinemia. In addition, 63 (57.3%) patients had EBV infection, but there was no statistical difference between the death group and the survival group within 30 days. By comparing the death vs. survival within 30 days of admission, we found statistically significant differences in neutrophil counts, serum sodium counts, and splenomegaly between the two groups (*p *= 0.019, *p *= 0.022, and *p *= 0.031, respectively). There were no differences in other laboratory parameters between groups.

**Table 1:** Demographic and clinical characteristics at presentation of 110 HLH patients according to survival groups.

	Total (*n* = 110)	Survived (*n* = 76)	Died (*n* = 34)	*P*, survived vs. Died^a^
Male^b^	62.0 (56.4)	41.0 (53.9)	21.0 (61.8)	0.534
Age (month)	44.0 (23.0–100.5)	45.0 (25.0–102.0)	47.0 (17.5–105.5)	0.589
Weight	15.0 (11.0–24.6)	15.0 (12.0–26.4)	14.0 (9.6–24.3)	0.445
Laboratory findings				
WBC (×10^9^/L)	2.7 (1.3–5.7)	2.3 (1.3–5.3)	3.7 (1.4–7.3)	0.395
ANC (×10^9^/L)	1.0 (0.4–2.2)	0.9 (0.3–1.7)	1.5 (0.7–3.9)	**0**.**019**
PLT (×10^9^/L)	51.0 (30.0–103.5)	54.0 (33.0–103.0)	46.0 (19.8–106.5)	0.158
Hg (g/L)	90.0 (73.0–98.25)	92.0 (74.0–98.0)	82.5 (69.8–104.8)	0.398
T-Bil (mmol/L)	12.9 (5.0–45.1)	11.4 (3.9–36.4)	17.0 (5.8–70.0)	0.132
LDH (U/L)	2150.0 (1589.5–4126.3)	2189.0 (1393.0–5095.0)	2150.0 (1704.0–3577.0)	0.618
AST (U/L)	268 (138.3–647.3)	270.0 (159.0–635.0)	287.5 (116.0–665.3)	0.882
ALT (U/L)	157.5 (72.0–341.8)	158.0 (73.0–337.0)	135.5 (63.5–356.8)	0.550
BUN (mmol/L)	4.0 (2.8–5.7)	3.7 (2.8–5.2)	4.6 (2.6–6.6)	0.337
Scr (mmol/L)	32.5 (25.0–44.0)	32.0 (25.0–45.0)	34.0 (25.0–44.0)	0.851
Serum calcium (mmol/L)	2.0 (1.9–2.1)	2.0 (1.9–2.1)	1.9 (1.8–2.1)	0.301
Serum sodium (mmol/L)	133.0 (130.0–135.6)	133.5 (131.1–136.2)	132.4 (128.2–134.6)	**0**.**022**
Tg (mmol/L)	3.1 (2.2–4.7)	3.1 (2.2–4.4)	3.3 (1.8–5.0)	0.974
HDL-C (mmol/L)	0.2 (0.1–0.4)	0.2 (0.1–0.4)	0.2 (0.1–0.4)	0.051
LDL-C (mmol/L)	1.1 (0.7–1.6)	1.2 (0.7–1.7)	0.9 (0.5–1.4)	0.166
Fibrinogen level (mg/dl)	117.5 (75.8–201.8)	123.0 (82.0–204.0)	107.0 (55.0–202.0)	0.284
CRP (mg/L)	15.5 (6.0–43.5)	15.0 (6.0–38.0)	28.7 (6.0–90.0)	0.182
Ferritin level (ng/ml)	5689.9 (1516.8–16500.0)	4816.5 (1471.3–16500.0)	8088.3 (1971.8–16500.0)	0.416
Clinical findings				
Hepatomegaly^b^	105.0 (95.5)	71 (93.4)	34 (100.0)	0.321
Splenomegaly^b^	90.0 (81.8)	58.0 (76.3)	32.0 (94.1)	**0**.**031**
EBV infection	63.0 (57.3)	45.0 (59.2)	18 (52.9)	0.677
sHLH^b^	96.0 (87.3)	63.0 (82.9)	33.0 (97.1)	0.06
Temperature >37.5°C^b^	109.0 (99.1)	75.0 (98.7)	34 (100.0)	1.000
Outcome				
ICU admission^b^	109.0 (99.1)	75.0 (98.7)	34.0 (100.0)	1.000

HLH, hemophagocytic lymphohistiocytosis; WBC, white blood cell count; PLT, platelet; Hg, Hemoglobin level; T-Bil, Total bilirubin level; LDH, lactate dehydrogenase level; AST, Aspartate aminotransferase level; ALT, Alanine transaminase level; BUN, Blood urea nitrogen level; Scr, Serum creatinine level; Tg, Triglycerides; HDL-C, High-density lipoprotein level; LDL-C, low-density lipoprotein-cholesterol level; CRP, C-reactive protein level; sHLH, secondary HLH.

Values are expressed as medians and interquartile ranges (IQRs).

^a^ By Mann–Whitney *U* test or Fisher's exact text.

^b^ Values are the number (%) of patients.

Table 2 is the information about the complications of our patients. Among them, the most common complication was coagulation disorder, which was diagnosed in 86 (78.2%) patients. There were statistically significant differences in MODS, respiratory failure, heart failure, acute liver failure, AKI, electrolyte disturbance, hypoproteinemia, and hyperlactatemia between the two groups in terms of death and survival within 30 days of admission (*p* = 0.011, *p *= 0.001, *p *< 0.001, *p *= 0.009, *p *< 0.001, *p *= 0.012, *p *= 0.004 and *p *= 0.011, respectively). There were no statistically significant differences in ARDS, sepsis, pneumonia, and liver dysfunction between the two groups.

**Table 2:** Statistics of complications in 110 patients with HLH according to survival groups.

	Total (*n* = 110)	Survived (*n* = 76)	Died (*n* = 34)	*P*, survived vs. Died^a^
MODS^b^	24.0 (21.8)	11.0 (14.5)	13.0 (38.2)	**0**.**011**
Respiratory Failure^b^	27.0 (24.5)	11.0 (14.5)	16.0 (47.1)	**0**.**001**
Heart Failure^b^	15.0 (13.6)	4.0 (5.3)	11.0 (32.4)	**<0**.**001**
Acute liver Failure^b^	10.0 (9.1)	3.0 (3.9)	7.0 (20.6)	**0**.**009**
AKI^b^	9.0 (8.2)	1.0 (1.3)	8.0 (23.5)	**<0**.**001**
ARDS^b^	10.0 (9.1)	2.0 (2.6)	8.0 (23.5)	0.001
Sepsis^b^	66.0 (60.0)	42.0 (55.3)	24.0 (70.6)	0.146
Coagulation dysfunction^b^	86.0 (78.2)	57.0 (75.0)	29.0 (85.3)	0.319
Pneumonia^b^	93.0 (84.5)	62.0 (81.6)	31.0 (91.2)	0.260
Electrolyte disturbances^b^	79.0 (71.8)	49.0 (64.5)	30.0 (88.2)	**0**.**012**
Hypoproteinemia^b^	51.0 (46.4)	28.0 (36.8)	23.0 (67.6)	**0**.**004**
Abnormal liver function^b^	80.0 (72.7)	59.0 (77.6)	21.0 (61.8)	0.106
Hyperlactatemia^b^	32.0 (29.1)	16.0 (21.1)	16.0 (47.1)	**0**.**011**

HLH, hemophagocytic lymphohistiocytosis; MODS, Multiple organ dysfunction Failure; AKI, Acute kidney injury; ARDS, Acute respiratory distress syndrome.

Values are expressed as medians and interquartile ranges (IQRs).

^a^ By Fisher's exact text.

^b^ Values are the number (%) of patients.

*p*-value <0.05 was considered significant and is highlight in bold.

Gene mutations were found in only 14 of the 110 patients during hospitalization (Supplementary Table S1), including 8 (57.1%) with UNC13D mutation, 4 (28.6%) with STXBP2 mutation, and 2 (14.3%) with PRF1 mutation. Notably, in our cohort, there was no statistically significant difference in sHLH diagnosis rates between the group that died within 30 days of admission and the group that survived (*p *= 0.06). As can be seen from Table 3, most patients were treated with antibiotics [109 (99.1%)] and dexamethasone [91 (82.7%)]. Continuous renal replacement therapy (CRRT), plasma exchange (PE), etoposide, dexamethasone, and assisted ventilation were statistically different between groups of deaths and survivors within 30 days of admission (*p *< 0.001, *p *= 0.031, *p *= 0.007, *p *= 0.002 and *p *< 0.001, respectively). Among them, the dose of etoposide in the survival group was 130.00 mg/m^2^ (IQR 116.17–145.60), the reported dose of liquid in the death group was 120.00 mg/m^2^ (IQR 108.55–128.00), and there was no significant difference in the dose of etoposide between the two groups (*p *= 0.205). There were no differences between groups in other therapies.

**Table 3:** Statistics of therapeutic strategies in 110 patients with HLH according to survival groups.

	Total (*n* = 110)	Survived (*n* = 76)	Died (*n* = 34)	*P*, survived vs. Died^a^
CRRT^b^	13.0 (11.8)	3.0 (3.9)	10.0 (29.4)	**<0**.**001**
PE^b^	19.0 (17.3)	9.0 (11.8)	10.0 (29.4)	**0**.**031**
Etoposide	55.0 (50.0)	45.0 (59.2)	10.0 (29.4)	**0**.**007**
Etoposide dose (mg/m^2^)	128.28 (114.55–145.22)	130.00 (116.17–145.60)	120.00 (108.55–128.00)	0.205
Dexamethasone^b^	91.0 (82.7)	69.0 (90.8)	22.0 (64.7)	**0**.**002**
Cyclosporine	33.0 (30.0)	27.0 (35.5)	6.0 (17.6)	0.073
Intravenous Immunoglobulin^b^	66.0 (60.0)	47.0 (61.8)	19.0 (55.9)	0.674
Antibiotics^b^	109.0 (99.1)	75.0 (98.7)	34.0 (100)	1.000
Assisted ventilation^b^	38.0 (34.5)	15.0 (19.7)	23.0 (67.6)	**<0**.**001**

HLH, hemophagocytic lymphohistiocytosis; CRRT, Continuous renal replacement therapy; PE, Plasma exchange.

Values are expressed as medians and interquartile ranges (IQRs).

^a^ By Fisher's exact text.

^b^ Values are the number (%) of patients.

*p-*value <0.05 was considered significant and is highlight in bold.

### Multivariable Logistic regression analysis is used for HLH

As shown in Table 4, multivariable logistic regression analysis was used to predict risk factors for death within 30 days of admission to HLH patients. These include neutrophil count, serum sodium, respiratory failure, heart failure, acute liver failure, AKI, splenomegaly, hypoproteinemia, hyperlactatemia, CRRT, PE, etoposide, dexamethasone, and assisted ventilation. Multivariable logistic regression models do not include electrolyte disturbances because they are strongly related to electrolyte disturbances. At the same time, the model does not include MODS, because it is strongly associated with complications of organ injury such as respiratory failure and heart failure.

**Table 4:** Multivariable logistic regression analysis for risk factors of death in HLH patients.

	*β*	SE	Walds	*p*-value	OR	95%CI for OR
ANC	0.029	0.065	0.199	0.655	1.030	0.906–1.171
Serum sodium	−0.083	0.061	1.829	0.176	0.921	0.817–1.038
Respiratory Failure	−0.538	0.955	0.317	0.573	0.584	0.090–3.797
Heart Failure	2.594	1.062	5.972	**0** **.** **015**	13.389	1.671–107.256
Acute liver Failure	−0.568	1.126	0.255	0.614	0.567	0.062–5.146
AKI	2.311	1.842	1.574	0.210	10.087	0.273–373.154
Splenomegaly	1.496	1.058	1.999	0.157	4.463	0.561–35.489
Hypoproteinemia	1.577	0.678	5.410	**0**.**020**	4.841	1.282–18.288
Hyperlactatemia	0.357	0.678	0.277	0.598	1.429	0.378–5.398
CRRT	−0.368	1.285	0.082	0.775	0.692	0.056–8.597
PE	0.369	1.025	0.129	0.719	1.446	0.194–10.785
Etoposide	−0.963	0.674	2.041	0.153	0.382	0.102–1.431
Dexamethasone	−1.249	0.884	1.995	0.158	0.287	0.051–1.623
Assisted ventilation	1.556	0.854	3.323	0.068	4.742	0.890–25.277
Interpret	8.282	8.186	1.024	0.312	3952.558	

HLH, hemophagocytic lymphohistiocytosis; AKI, Acute kidney injury; OR, Odds Ratio; CI, confidence interval.

*p*-value <0.05 was considered significant and is highlight in bold.

**Figure 2:**
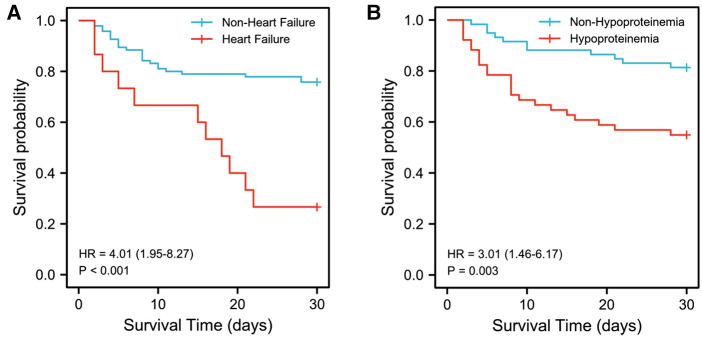
Kaplan-Meier survival estimates of survival rate. (**A**) Kaplan-Meier survival estimates of survival rate in 110 patients with or without heart failure. (**B**) Kaplan-Meier survival estimates of survival rate in 110 patients with or without hypoproteinemia.

**Figure 3:**
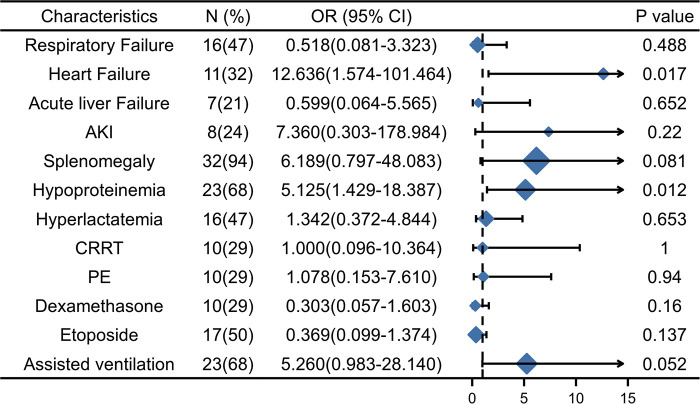
The subgroup analyses of overall survival. AKI, Acute kidney injury, CRRT, Continuous renal replacement therapy; PE, Plasma exchange.

The results of multivariable logistic regression analysis showed that heart failure (OR = 13.389, 95% CI, 1.671–107.256, *p *= 0.015) and hypoproteinemia (OR = 4.841, 95% CI, 1.282–18.288, *p *= 0.020) were significantly associated with the increase in early HLH deaths. Other neutrophil counts, serum sodium, respiratory failure, AKI, acute liver failure, splenomegaly, hyperlactatemia, CRRT, PE, etoposide, dexamethasone, and assisted ventilation were not significantly associated with increased early mortality (*p *= 0.655, *p *= 0.176, *p *= 0.573, *p *= 0.210, *p *= 0.614, *p *= 0.157, *p *= 0.598, *p *= 0.775, *p *= 0.153, *p *= 0.719, *p *= 0.158, and *p *= 0.068, respectively). Based on the risk factors (heart failure and hypoproteinemia) derived from a multivariable logistic regression model, we plotted the 30-day survival curves of patients with HLH after admission (Figure 2). In addition, we performed subgroup analyses by respiratory failure (yes vs. no), heart failure (yes vs. no), acute liver failure (yes vs. no), AKI (yes vs. no), splenomegaly (yes vs. no), hypoproteinemia (yes vs. no), hyperlactic acidemia (yes vs. no), CRRT (yes vs. no), PE (yes vs. no), etoposide (yes vs. no), dexamethasone (yes vs. no), and assisted ventilation (yes vs. no), as shown in Figure 3, and we came to the same conclusion. Heart failure (OR = 12.636, 95% CI, 1.574–101.464, *p* = 0.017) and hypoproteinemia (OR = 5.125, 95% CI, 1.429–18.387, *p* = 0.012) were significantly associated with an increase in early HLH deaths.

## Discussion

In our study, early mortality in HLH children remained high. In order to describe HLH patients who died shortly after hospitalization and to identify risk factors associated with early death, we described and analyzed the demographic information and clinical features of 110 children with HLH. The 30-day OS for children with HLH was 69.1%. The established risk factors for 30-day mortality were heart failure and hypoproteinemia, of which heart failure was strong risk factors (*p* < 0.05 and OR > 10).

In our patients, 30-day OS was 69.1%. We identified several other large case families of pediatric HLH patients in Chinese populations with a 30-day OS of 66%–86% (18–21). Our 30-day OS is similar to these results. Reports of early mortality in HLH patients in other regions are equally striking. Studies suggest that in one HLH patient with hypofibrinogenemia, the OS was 73.7% at 28 days (22). Strikingly, up to 109 (99.1%) of our case cohorts were admitted to the ICU, meaning that without timely and effective treatment, patients would be life-threatening. Therefore, it will be of great significance to analyze the risk factors for early death of patients with HLH, stratify the patients, and adopt timely and effective treatment strategies in a targeted manner. In addition, although previous reports suggest improved long-term survival in patients with HLH (14), it is still not optimistic. Therefore, this cohort follow-up of ours is continuing in order to characterize and analyze the long-term survival rates of HLH patients at 2 and 5 years.

A common complication was coagulation disorders, up to 86 cases (78.2%). In addition, because both sepsis and HLH are hyper-inflammatory syndromes and there is an overlap in diagnostic criteria between the two (23, 24), up to 66 (60%) of children are diagnosed with sepsis. This is consistent with previous research. It is difficult to explain the relationship between sepsis and HLH, and more sophisticated diagnostic indicators may be needed in the future to distinguish between the two diseases. Notably, there was no statistically significant difference in the incidence of sepsis between the two groups that died within 30 days of our and those who survived.

Our investigation identified clinical features that are significantly associated with early mortality, which is consistent with previous findings (25). Heart failure and hypoproteinemia are risk factors for increased mortality within 30 days. Because HLH can rapidly develop into MODS, if it is not treated in a timely and effective manner, it will endanger the lives of patients (26). Our study is consistent with a higher incidence of MODS (38.2% vs. 14.5%) in patients who died within 30 days than in surviving patients. However, because MODS is strongly associated with a variety of diseases such as heart failure, respiratory failure, and AKI, our final logistic regression model did not include MODS. Nonetheless, MODS remains a risk factor for death in patients with HLH (27), and targeted treatment is needed to improve patient outcomes.

The incidence of EBV-HLH is high in East Asian countries (28). The vast majority of our cohort is sHLH, and 57.3% of patients are positive for EBV infection Table 1, and other causes of sHLH are tumors, autoimmune diseases, or immunodeficiency. Our study shows that EBV infection has no difference between the survival and death groups and is not a risk factor for early death, which is similar to the previous conclusion that patients with EBV-HLH tend to have good outcomes when treated in a timely manner (29). In addition, in our cohort, sHLH did not affect the outcome of early treatment (*p *= 0.06) (Table 1). The therapeutic strategy for HLH is to support treatment and control of the overactivated immune system. It mainly includes immunosuppressive therapy (dexamethasone) and etoposide, as well as supportive therapy for complications. In our cohort, patients were treated with HLH-2004 therapy. Among them, 82.7% of patients were treated with dexamethasone, 50% were treated with etoposide, and 30% were treated with cyclosporine. It is worth noting that the rate of etoposide use in the death group is lower than that in the survival group (*p *= 0.007), which is related to the more serious complications (acute liver injury, acute renal failure) in the death group, because these complications are contraindications to etoposide use. Due to the different rates of etoposide use in the two groups, we calculated the dose of etoposide in both groups. In our cohort, etoposide was used once a week for the first 8 weeks at a dose of 128.28 mg/m^2^ (IQR 114.55–145.22), and the dose was flexibly adjusted according to the patient's liver and kidney function. There was no significant difference in etoposide use between the survival group and the death group within 30 days of admission (*p *= 0.205), and the dose was not low. In the future, clinicians may need to find an optimal balance between contraindications to etoposide use and effective treatment in patients with HLH. By multivariate regression analysis, we found that the use of etoposide was not associated with a better outcome (OR). In addition, due to other complications, we gave patients CRRT (to rescue AKI), PE [to replace of cytokines and correct of coagulation disorders by fresh frozen plasma (FFP)], intravenous Immunoglobulin (to improve immunity), antibiotics (to fight infection), and assisted ventilation (to rescue respiratory failure).

The strength of this study is that it includes a large number of patients with rare diseases and describes in detail the demographic characteristics, clinical features, complications and treatment strategies of the patients. Our study provides a reference for identifying and stratifying high-risk patients for HLH and further targeted treatment strategies. Our study has some flaws, starting with the fact that this study is a single-center, retrospective study that may have selection bias and missing information and affect patient features and clinical data. Second, the vast majority of patients in our cohort are sHLH, so the findings of the study may be more appropriate for patients with sHLH. In addition, soluble CD25 is not routinely tested and may be missed diagnoses. Therefore, more accurate description and analysis of multicenter, prospective, randomized controlled studies with larger sample sizes are needed in the future.

## Conclusions

We identified risk factors that are strongly associated with early mortality in pediatric patients with HLH. This may help clinicians stratify HLH patients and develop targeted treatment strategies. More research is needed to explore the best treatment strategies for patients with HLH to reduce early mortality in patients with HLH.

## Data Availability

The raw data supporting the conclusions of this article will be made available by the authors, without undue reservation.

## References

[1] Zhang L Zhou J Sokol L. Hereditary and acquired hemophagocytic lymphohistiocytosis. Cancer Control. (2014) 21(4):301–12. 10.1177/107327481402100406 25310211

[2] La Rosée P Horne A Hines M von Bahr Greenwood T Machowicz R Berliner N Recommendations for the management of hemophagocytic lymphohistiocytosis in adults. Blood. (2019) 133(23):2465–77. 10.1182/blood.2018894618 30992265

[3] Fardet L Lambotte O Meynard J-L Kamouh W Galicier L Marzac C Reactive haemophagocytic syndrome in 58 HIV-1-infected patients: clinical features, underlying diseases and prognosis. AIDS. (2010) 24(9):1299–306. 10.1097/QAD.0b013e328339e55b 20559036

[4] Fałkowska A Prądzyńska K Drabko K. Difficult balance between EBV treatment and posttransplant immunosuppression: a successful transplant in a child with recurrent epstein-barr virus–induced hemophagocytic lymphohistiocytosis. Transplant Proc. (2021) 53(6):2035–9. 10.1016/j.transproceed.2021.03.044 33933286

[5] Strippoli R Caiello I De Benedetti F. Reaching the threshold: a multilayer pathogenesis of macrophage activation syndrome. J Rheumatol. (2013) 40(6):761–7. 10.3899/jrheum.121233 23588947

[6] Henter J-I Horne A Aricó M Egeler RM Filipovich AH Imashuku S HLH-2004: diagnostic and therapeutic guidelines for hemophagocytic lymphohistiocytosis. Pediatr Blood Cancer. (2007) 48(2):124–31. 10.1002/pbc.21039 16937360

[7] Cron RQ Davi S Minoia F Ravelli A. Clinical features and correct diagnosis of macrophage activation syndrome. Expert Rev Clin Immunol. (2015) 11(9):1043–53. 10.1586/1744666x.2015.1058159 26082353

[8] Deiva K Mahlaoui N Beaudonnet F de Saint Basile G Caridade G Moshous D CNS Involvement at the onset of primary hemophagocytic lymphohistiocytosis. Neurology. (2012) 78(15):1150–6. 10.1212/WNL.0b013e31824f800a 22422896

[9] Horne A Trottestam H Aricò M Egeler RM Filipovich AH Gadner H Frequency and spectrum of central nervous system involvement in 193 children with haemophagocytic lymphohistiocytosis. Br J Haematol. (2008) 140(3):327–35. 10.1111/j.1365-2141.2007.06922.x 18076710

[10] Tateishi Y Oda S Sadahiro T Nakamura M Hirayama Y Abe R Continuous hemodiafiltration in the treatment of reactive hemophagocytic syndrome refractory to medical therapy. Transfus Apher Sci. (2009) 40(1):33–40. 10.1016/j.transci.2008.11.001 19097821

[11] Henter JI Arico M Egeler RM Elinder G Favara BE Filipovich AH HLH-94: a treatment protocol for hemophagocytic lymphohistiocytosis. Med Pediatr Oncol. (1997) 28(5):342–7. 10.1002/(Sici)1096-911x(199705)28:5<342::Aid-Mpo3>3.0.Co;2-H 9121398

[12] RB S, AHT R-S. Histiocytic medullary reticulosis. Lancet. (1939) 234:194–8. 10.1016/S0140-6736(00)61951-7

[13] Park H-S Kim D-Y Lee J-H Lee J-H Kim S-D Park Y-H Clinical features of adult patients with secondary hemophagocytic lymphohistiocytosis from causes other than lymphoma: an analysis of treatment outcome and prognostic factors. Ann Hematol. (2011) 91(6):897–904. 10.1007/s00277-011-1380-3 22147006

[14] Yoon J-H Park S-S Jeon Y-W Lee S-E Cho B-S Eom K-S Treatment outcomes and prognostic factors in adult patients with secondary hemophagocytic lymphohistiocytosis not associated with malignancy. Haematologica. (2019) 104(2):269–76. 10.3324/haematol.2018.198655 30213834 PMC6355492

[15] Filipovich AH. Hemophagocytic lymphohistiocytosis (HLH) and related disorders. Hematology. (2009) 2009(1):127–31. 10.1182/asheducation-2009.1.127 20008190

[16] Ponikowski P Voors AA Anker SD Bueno H Cleland JGF Coats AJS ESC Guidelines for the diagnosis and treatment of acute and chronic heart failure. Eur Heart J. (2016) 37(27):2129–200. 10.1093/eurheartj/ehw128 27206819

[17] Palevsky PM Liu KD Brophy PD Chawla LS Parikh CR Thakar CV KDOQI US commentary on the 2012 KDIGO clinical practice guideline for acute kidney injury. Am J Kidney Dis. (2013) 61(5):649–72. 10.1053/j.ajkd.2013.02.349 23499048

[18] Li X Yan H Luo T Xiao Z Gong L Huang J Fulfillment status of hypertriglyceridemia and hypofibrinogenemia in children with hemophagocytic lymphohistiocytosis and risks of multiple organ dysfunction syndrome and early mortality. Orphanet J Rare Dis. (2022) 17(1):161. 10.1186/s13023-022-02315-8 35410268 PMC8996201

[19] Li X Yan H Zhang X Huang J Xiang S-T Yao Z Elevated serum myoglobin levels at hospital admission and the risk of early death among patients with hemophagocytic lymphohistiocytosis: evidence from 155 pediatric patients. Ann Hematol. (2020) 99(5):963–71. 10.1007/s00277-020-03980-0 32221652

[20] Bin Q Gao J-H Luo J-M. Prognostic factors of early outcome in pediatric hemophagocytic lymphohistiocytosis: an analysis of 116 cases. Ann Hematol. (2016) 95(9):1411–8. 10.1007/s00277-016-2727-6 27307280

[21] Luo Z-B Chen Y-Y Xu X-J Zhao N Tang Y-M. Prognostic factors of early death in children with hemophagocytic lymphohistiocytosis. Cytokine. (2017) 97:80–5. 10.1016/j.cyto.2017.03.013 28582648

[22] Signoff JK Fitzgerald JC Teachey DT Balamuth F Weiss SL. Hypofibrinogenemia is associated with poor outcome and secondary hemophagocytic lymphohistiocytosis/macrophage activation syndrome in pediatric severe sepsis*. Pediatr Crit Care Med. (2018) 19(5):397–405. 10.1097/pcc.0000000000001507 29470247 PMC9630256

[23] Hines MR von Bahr Greenwood T Beutel G Beutel K Hays JA Horne A Consensus-based guidelines for the recognition, diagnosis, and management of hemophagocytic lymphohistiocytosis in critically ill children and adults. Crit Care Med. (2021) 50(5):860–72. 10.1097/ccm.0000000000005361 34605776

[24] Strauss R Neureiter D Westenburger B Wehler M Kirchner T Hahn EG. Multifactorial risk analysis of bone marrow histiocytic hyperplasia with hemophagocytosis in critically ill medical patients—a postmortem clinicopathologic analysis. Crit Care Med. (2004) 32(6):1316–21. 10.1097/01.Ccm.0000127779.24232.15 15187513

[25] Li X Yan H Zhang X Huang J Xiang S-T Yao Z Clinical profiles and risk factors of 7-day and 30-day mortality among 160 pediatric patients with hemophagocytic lymphohistiocytosis. Orphanet J Rare Dis. (2020) 15(1):229. 10.1186/s13023-020-01515-4 32867836 PMC7456759

[26] Buyse S Teixeira L Galicier L Mariotte E Lemiale V Seguin A Critical care management of patients with hemophagocytic lymphohistiocytosis. Intensive Care Med. (2010) 36(10):1695–702. 10.1007/s00134-010-1936-z 20532477

[27] Eloseily EM Weiser P Crayne CB Haines H Mannion ML Stoll ML Benefit of anakinra in treating pediatric secondary hemophagocytic lymphohistiocytosis. Arthrit Rheumatol. (2019) 72(2):326–34. 10.1002/art.41103 31513353

[28] Koh K-N Im HJ Chung N-G Cho B Kang HJ Shin HY Clinical features, genetics, and outcome of pediatric patients with hemophagocytic lymphohistiocytosis in Korea: report of a nationwide survey from Korea Histiocytosis Working Party. Eur J Haematol. (2015) 94(1):51–9. 10.1111/ejh.12399 24935083 PMC7163615

[29] Imashuku S TT Tauchi H Ishida Y Otoh Y Sawada M Tanaka H Longitudinal follow-up of patients with Epstein-Barrvirus-associated hemophagocytic lymphohistiocytosis. Haematologica. (2004) 89(2):183–8. 15003893

