# Supplementary material for: Risk factors of early death in pediatric hemophagocytic lymphohistocytosis: Retrospective cohort study
Source: Front Pediatr. 2022 Oct 21;10:1031432. doi: 10.3389/fped.2022.1031432 (PMC9634417; doi:10.3389/fped.2022.1031432)
Supplement: Supplementary file 1 [file Table1.docx]

**Supplementary table 1.**

HLH genetic testing results

| Test result | N (%) |
| --- | --- |
| All HLH gene mutation | 14.0(12.7) |
| UNC13D | 8.0(57.1) |
| STXBP2 | 4.0(28.6) |
| PRF1 | 2.0(14.3) |

HLH=hemophagocytic lymphohistiocytosis.
